# Potential utility of targeted Nanopore sequencing for improving etiologic diagnosis of bacterial and fungal respiratory infection

**DOI:** 10.1186/s13000-020-00960-w

**Published:** 2020-04-27

**Authors:** Wai Sing Chan, Chun Hang Au, Sau Man Leung, Dona N. Ho, Elaine Yue Ling Wong, Man Yan To, Man Kin Ng, Tsun Leung Chan, Edmond Shiu Kwan Ma, Bone Siu Fai Tang

**Affiliations:** Department of Pathology, Hong Kong Sanatorium & Hospital, Hong Kong, China

**Keywords:** 16S, Bacterial pathogen, Etiologic diagnosis, Fungal pathogen, ITS, Nanopore, Opportunistic infection, Pneumonia, Respiratory infection

## Abstract

**Background:**

Diversified etiology of lower respiratory tract infection renders diagnosis challenging. The mainstay microbial culture is time-consuming and constrained by variable growth requirements. In this study, we explored the use of Nanopore sequencing as a supplementary tool to alleviate this diagnostic bottleneck.

**Methods:**

We developed a targeted Nanopore method based on amplification of bacterial 16S rRNA gene and fungal internal transcribed spacer region. The performance was compared with routine infectious disease workups on 43 respiratory specimens.

**Results:**

Nanopore successfully identified majority of microbes (47/54, 87.04%) and 7 possible pathogens not detected by routine workups, which were attributable to the content of microbiological investigations (*n* = 5) and negative culture (*n* = 2). The average sequencing time for first target reads was 7 min (1–43 min) plus 5 h of pre-sequencing preparation.

**Conclusions:**

The Nanopore method described here was rapid, economical and hypothesis-free, which might provide valuable hints to further microbiological follow-up for opportunistic pathogens missed or not detectable by conventional tests.

## Introduction

Lower respiratory tract infections (LRTI) are a leading cause of morbidity and mortality worldwide [1]. As guided by clinical and epidemiological factors, microbiological investigations may be carried out to determine the etiology, especially for community-acquired pneumonia (CAP) with moderate and high severity [2, 3]. Nevertheless, CAP pathogens are diversified which render etiologic diagnosis challenging. The mainstay microbial culture is time-consuming and may only identify a pathogen up to 40% of cases due to variable growth requirements [4]. Multiplex polymerase chain reaction (PCR) assays have been demonstrated useful for improving pathogen detection from lower respiratory specimens [5, 6], yet the targets are confined to key bacteria which may only represent the tip of an iceberg.

Recently, next-generation sequencing (NGS) facilitates a universal approach for pathogen detection, which may revolutionize infectious disease diagnostics [7]. In particular, Nanopore technology provides the speed that can shorten sample-to-answer time, which is important for the severely ill. From literature, several research groups explored the use of Nanopore sequencing for pneumonia diagnosis, utilizing both metagenomic and targeted approaches [8–11]. In this study, we adopted the targeted approach because it enables higher depth of microbial reads and thus allowing more room for multiplexing which significantly lowers the cost. We developed a targeted Nanopore method for simultaneous detection of bacterial and fungal pathogens, and compared its performance with routine infectious disease workups on archived respiratory specimens harboring diversified microbial species.

## Methods

### Specimens

The specimens were selected based on microbial diversity. The 43 respiratory specimens were collected between May 2016 and September 2019 in Department of Pathology, Hong Kong Sanatorium & Hospital. These comprised 34 lower and 9 upper respiratory specimens, and the details are summarized in Table 1.

**Table 1:** Details of clinical specimens and results of Nanopore sequencing

Patient	Specimen		Routine infectious disease workups		Nanopore sequencing		Possible pathogens^#^ (abundance)	Remarks
		N^*^	Test	Result^§^	Time^†^	Abundance^¶^		
1	BAL	5	Culture	*C. albicans* *C. glabrata*	4 min 4 min	61% 22%	/	/
2	Sputum	2	Culture	*Candida* sp.^(M)^	4 min	61%	**/**	/
3	Sputum	6	Culture	*S. pyogenes*^(H)^	9 min	59%	/	/
4	Sputum	5	Culture	*H. influenzae*^(H)^	22 min	41%	/	/
5	BAL	7	Culture Microscopy	*C. albicans*^(M)^ *P. jirovecii*	5 min 5 min	93% 0.1%	/	/
6	Sputum	5	Culture	*S. pneumoniae*^(H)^	5 min	6%	/	*S. pneumoniae* antigen (urine): negative
7	BAL	7	Culture PCR	*C. albicans*^(S)^ *L. pneumophila*	5 min 4 min	98% 98%	/	/
8	BAL	7	Culture	*C. albicans*^(S)^	2 min	85%	/	/
9	NPS	1	PCR	*B. parapertussis*	32 min	0.2%	/	/
10	Sputum	3	Culture	*M. abscessus*	27 min	0.3%	**/**	AFB smear: negative
11	BAL	5	Culture	*M. avium* complex *Raoultella* sp.^(S)^ *S. aureus*^(S)^	ND ND 2 min	ND ND 0.5%	/	AFB smear: negative
12	NPS	2	PCR Serum IgG	*C. pneumoniae*	2 min	0.8%	***S. pneumoniae*(80%)**	**Bacterial culture: not performed** **Consensus sequence showed 100% identity to*S. pneumoniae***
13	Sputum	3	PCR	*M. pneumoniae*	10 min	99%	/	/
14	NPA	3	Culture	*C. pseudodiphtheriticum*^(H)^	4 min	4%	/	/
15	NPS	2	Culture	*M. catarrhalis*^(M)^	16 min	98%	/	/
16	Sputum	2	Culture	*M. catarrhalis*^(M)^	13 min	98%	/	/
17	Lung Bx	5	Culture	*C. neoformans*	3 min	67%	/	/
18	Sputum	4	PCR	*P. jirovecii*	12 min	97%	***L. pneumophila*(11%)**	***L. pneumophila*PCR: not performed** **Consensus sequence showed 99% identity to*L. pneumophila*** *P. jirovecii* DNA: 3.13 × 10^6^ copies/mL
19	Sputum	6	PCR Culture Culture	*P. jirovecii* *S. aureus*^(S)^ *C. albicans*^(S)^	8 min 43 min 8 min	0.001% 0.1% 74%	/	*P. jirovecii* DNA: 1.77 × 10^6^ copies/mL
20	NPS	2	PCR	*C. pneumoniae*	1 min	0.03%	/	/
21	BAL	2	NGS	*R. mucilaginosa*	1 min	67%	**/**	**Culture-negative*R. mucilaginosa*** **Shotgun NGS by MiSeq** **Consensus sequence showed 99% identity to*R. mucilaginosa***
22	BW	5	PCR Culture	*M. tuberculosis* complex *C. dubliniensis*	9 min 3 min	0.01% 2%	**/**	AFB smear: positive
23	NPS	1	PCR	*B. pertussis*	40 min	0.02%	/	/
24	BAL	7	Culture Culture PCR	*C. albicans* *P. kudriavzevii* *P. jirovecii*	1 min ND 1 min	31% ND 33%	***L. wadei*(64%)**	**Culture-negative*L. wadei*** **Consensus sequence showed 99% identity to*L. wadei*** *P. jirovecii* DNA: 3.43 × 10^7^ copies/mL
25	ETA	4	Culture	*Candida* sp.^(S)^	ND	ND	/	/
26	BAL	4	PCR	*P. jirovecii*	2 min	50%	**/**	*P. jirovecii* DNA: 8.82 × 10^4^ copies/mL
27	TA	2	Culture	*C. dubliniensis*^(S)^	1 min	90%	**/**	/
28	NPS	2	PCR Serum IgM	*C. pneumoniae*	1 min	84%	/	/
29	BW	3	Culture	*S. maltophilia*^(S)^ *C. albicans*^(S)^ *C. glabrata*^(S)^	1 min 1 min 1 min	7% 51% 37%	/	/
30	Sputum	6	PCR	*M. pneumoniae*	1 min	65%	/	/
31	Sputum	7	PCR	*M. pneumoniae*	1 min	53%	/	/
32	BAL	6	PCR	*M. pneumoniae* *C. albicans*	2 min ND	1% ND	/	Fungus culture: negative
33	Sputum	5	PCR	*M. pneumoniae*	1 min	90%	/	/
34	BAL	7	Culture	*C. albicans*^(S)^	2 min	86%	/	Serum beta-D-glucan: negative
35	Sputum	6	Culture	*Burkholderia* sp.^(M)^	1 min	46%	/	/
36	Sputum	5	PCR	*M. pneumoniae*	1 min	12%	/	/
37	Sputum	7	Culture Urine Ag	*S. pneumoniae*^(M)^	3 min	0.6%	***C. albicans*(85%)**	**Fungus culture: not performed** **Consensus sequence showed 100% identity to*C. albicans***
38	Sputum	2	PCR	*P. jirovecii*	ND	ND	***C. striatum*(84%)** ***C. haemulonis*(70%)**	*P. jirovecii* DNA: < 760 copies/mL **Bacterial/ fungus culture: not performed** **Consensus sequence showed 99 and 100% identity to*C. striatum*and*C. haemulonis*, respectively.**
39	Sputum	2	PCR	*M. pneumoniae*	1 min	47%	/	/
40	NPS	1	PCR	*M. pneumoniae*	ND	ND	/	PCR Cp value = 37.06 (45 cycles)
41	NPS	1	PCR	*M. pneumoniae*	2 min	0.9%	/	/
42	Sputum	1	Culture	Normal flora	22 min	/	/	16S data revealed reads from normal flora dominated by *N. perflava* (19.55%) with *H. influenzae* colonization (1.68%). ITS sequencing: not performed
43	Sputum	6	Culture	Normal flora	87 min	/	/	16S data revealed reads from normal flora dominated by *L. buccalis* (46.08%). ITS data revealed no fungal reads. Fungus culture: negative

Summary

○ Average number of routine tests taken for bacterial/ fungal etiology: 4 (1–7)

○Average elapsed sequencing time of the first target read: 7 min (1–43 min; pre-sequencing preparation: 5 h)

○ Concordance with routine test results: 47/54 = 87.04%

○ Possible pathogens detected by Nanopore workflow: 7 from 6 patients

^*^Number of tests taken for bacterial/ fungal etiology

^§^Scanty^(S)^, moderate^(M)^ and heavy^(H)^ growth correspond to microbial growth up to primary, second and third sector of culture agar plate, respectively

^†^Elapsed Nanopore sequencing time of first target reads

^¶^Bacterial and fungal abundance were calculated separately

^#^Possible pathogens not detected by routine workups but identified by Nanopore sequencing

### Deoxyribonucleic acid (DNA) extraction and PCR

Standard laboratory practices were applied to minimize risk of infection and contamination. DNA extraction was performed using EMAG (bioMérieux, Marcy, I’Etoile, France) with blank lysis buffer as negative control. Bacterial 16S ribosomal ribonucleic acid (rRNA) gene and fungal internal transcribed spacer (ITS) region were amplified using universal primers 27F-1492R/ 533F-1100R and ITS5-ITS4, respectively, with human beta-globin gene as PCR inhibition control. Primer sequences and PCR conditions are described in Tables 2 and 3.

**Table 2:** PCR primers for bacterial 16S, fungal ITS and human beta-globin PCR

Primers	Sequences (5′ to 3′)	References
16S (27F)	AGAGTTTGATCMTGGCTCAG	[12]
16S (533F)	GTGCCAGCMGCCGCGGTAA	[13]
16S (1100R)	AGGGTTGCGCTCGTTG	[14]
16S (1492R)	TACGGYTACCTTGTTACGACTT	[12]
ITS5	GGAAGTAAAAGTCGTAACAAGG	[15]
ITS4	TCCTCCGCTTATTGATATGC	
Human beta-globin forward (BG-F)	GAAGAGCCAAGGACAGGTAC	[16]
Human beta-globin reverse (BG-R)	GGAAAATAGACCAATAGGCAG

**Table 3:** Master mix constituents and PCR conditions

16S PCR		
***Master mix preparation (for single reaction)***		
**Reagent**		**Volume/μL**
Platinum PCR SuperMix High Fidelity (Invitrogen)		20
27F/ 533F (10 μM)		1
1492R/ 1100R (10 μM)		1
DNA		5
***PCR conditions***		
**Temperature/**^**o**^**C**	**Time**	**No. of cycles**
94	2 min	1
94 55 68	30 s 30 s 2 min	30
68	7 min	1
12	Hold	/
ITS/ human beta-globin PCR		
***Master mix preparation (for single reaction)***		
**Reagent**		**Volume/μL**
10X PCR Buffer II (Applied Biosystems)		2.5
25 mM MgCl_2_ (Applied Biosystems)		1.5
AmpliTaq Gold DNA Polymerase (Applied Biosystems)		0.5
10 mM dNTPs (Roche)		0.5
Molecular grade water (Sigma)		10
ITS5/ BG-F (10 μM)		2.5
ITS4/ BG-R (10 μM)		2.5
DNA		5
***PCR conditions***		
**Temperature/**^**o**^**C**	**Time**	**No. of cycles**
95	10 min	1
95	15 s	
52	30 s	50
72	90 s	
72	7	1
12	Hold	/

### Nanopore sequencing library preparation

Nanopore sequencing libraries were prepared using Ligation Sequencing Kit 1D (SQK-LSK109) (Oxford Nanopore Technologies, Oxford, England) with or without PCR-free Native Barcoding Expansion Kit (EXP-NBD104/114) (Oxford Nanopore Technologies, Oxford, England). The libraries were loaded and sequenced on MinION flow cells (FLO-MIN106D R9.4.1) (Oxford Nanopore Technologies, Oxford, England) after quality control runs.

### Data analysis

MinKNOW version 2.0 was used for live basecalling. FASTQ files were analyzed on Nanopore EPI2ME platform with default minimum qscore of 7. Bacterial and fungal identification were facilitated via 16S and ‘What’s in my Pot?’ (WIMP) workflows, respectively. Reads assigned to all target and possible pathogens were counter-checked with National Center for Biotechnology Information (NCBI) basic local alignment search tool (BLAST), or re-analyzed by GAIA 2.0. For all possible pathogens, consensus sequences were built from BAM files using Unipro UGENE (version 1.29.0) and their similarity to reference genomes was determined. The elapsed sequencing time of first target reads was recorded. Relative abundance values of bacterial and fungal pathogens were calculated separately.

### Confirmation of possible pathogens

Presence of possible pathogens was further confirmed by PCR-sequencing using genus- or species-specific primers (Table 4).

**Table 4:** Genus- and species-specific primers for confirmation of possible pathogens

Primers	Sequences (5′ to 3′)	References
*S. pneumoniae* forward	AGGATAAGGAACTGCG	This study
*S. pneumoniae* reverse	CTTATTTTCTGACCTTTCA	
*L. wadei* forward	CCAAGCCAACCTTCAAAGCC	This study
*L. wadei* reverse	ACCTTGTTACGACTTCACCCC	
*C. albicans* forward	CTGCTGATATTACTGTTGGTTC	[17]
*C. albicans* reverse	CCACCAATACCAACGGTATC	
*Corynebacterium* forward	CACTGTGAGGTGGAGCGAAT	This study
*Corynebacterium* reverse	CCACCTTCGACAGCTCCCTA	
*C. haemulonis* forward	CGCATCGATGAAGAACGCAG	This study
*C. haemulonis* reverse	AGCGTAGACTTCACTGTGGC	

## Results

The results are summarized in Table 1. The first target reads were detected with average sequencing time of 7 min (1–43 min), plus 5 h of pre-sequencing preparation. Nanopore successfully identified majority of the microbes (47/54, 87.04%), and the false negatives were primarily due to low microbial burden, as reflected by negative smear (Patient 11), scanty or no microbial growth from culture (Patient 11, 25 and 32), and late cycle threshold values of real-time PCR assays (Patient 38 and 40).

Interestingly, Nanopore identified 7 possible pathogens from 6 patients, which were not detected by routine workups. The consensus sequences of these possible pathogens showed 99–100% identity to reference sequences, and their presence was confirmed by PCR-sequencing except for Patient 18 due to sample insufficiency. The 7 possible pathogens included common causes of typical and atypical pneumonia (*n* = 2: *Legionella pneumophila* and *Streptococcus pneumoniae*), as well as opportunistic pathogens (*n* = 5: *Candida albicans, Candida haemulonis, Corynebacterium striatum*, *Leptotrichia wadei* and *Rothia mucilaginosa*). These findings might be attributable to 2 factors. First, the routine workups did not include tests for microbes concerned (n = 5). Taking Patient 12 as an example, results of sputum PCR and serum immunoglobulin G test suggested *Chlamydia pneumoniae* infection, yet 80 and 0.8% of 16S reads were mapped to *S. pneumoniae* and *C. pneumoniae*, respectively. The former was more abundant and might have been isolated by culture, however, bacterial culture was not included in the workup. Another factor was negative culture (*n* = 2). For instance, in the bronchoalveolar lavage of Patient 24, majority of 16S reads (64%) were mapped to *L. wadei*, which might prefer anaerobic growth conditions and therefore not isolated from standard culture [18].

## Discussion

Our data revealed the heterogeneous nature of respiratory microbiome of the patients, which is hardly hinted by clinical signs and symptoms [19], but the content of microbiological investigation is often determined by this limited information. Considering the 6 patients with possible pathogens, half of the routine workups comprised 4–7 tests (average for all patients: 4). To a certain extent, the data revealed the bottleneck of etiologic diagnosis, that is, routine workups comprised a bundle of narrow-spectrum tests without targeting the culprit. Broad-spectrum, hypothesis-free NGS methods may contribute to improved etiologic diagnosis by supplementing the limitations of conventional tests and providing hints for further microbiological follow-up. A more complete picture on relative abundance of each microbial species may also help distinguish between causative agents and colonizers, especially in co-infection. Benefiting from the real-time sequencing by Nanopore technology, sample-to-answer time may be reduced to facilitate prompt pathogen-specific confirmatory work and treatment, which are essential for improved clinical outcome.

There are several diagnostic-grade molecular tests for pneumonia diagnosis, for example, the U.S. Food and Drug Administration (FDA)-approved BioFire FilmArray Pneumonia Panel and Explify Respiratory by ARUP Laboratories and IDbyDNA. The former identifies 33 targets from lower respiratory specimens in about 1 h, including 18 common LRTI bacteria with semi-quantitative results for 15 [20]. The latter works by metagenomic DNA and RNA sequencing (illumina NextSeq) enabling non-targeted detection of virtually any microbial targets within 48 h [21]. Our targeted Nanopore method is an intermediate between the two, in terms of speed (FilmArray > Nanopore > Explify) and detection spectrum (Explify > Nanopore > FilmArray). FilmArray appears to be a good option for first-line pathogen detection and Explify/ Nanopore may be a useful investigatory tool for culture- and FilmArray-negative opportunistic infections caused by rare pathogens. Considering the reagent cost, our Nanopore method is a competitive option among the 3. The per-sample reagent cost was about USD 68.87 for a 24-plex MinION run, and could be flexibly scaled for single sporadic cases on Flongle (USD 218.23) (Table 5). Nevertheless, as Nanopore technology is rapidly evolving, it is not easy to clinically validate a Nanopore-based assay at this moment due to requirements on ‘wet lab’ and bioinformatics analysis method standardization.

**Table 5:** Estimated reagent cost of Nanopore workflow (24-plex for MinION; 1 specimen for Flongle)

	Reagent cost/ service charge per specimen (USD)			
	**Nanopore**		**Send-out for** **pneumonia testing**	**FDA-cleared pneumonia panel** **(33 targets)**
	**MinION** **(23 specimens + 1 NC)**	**Flongle** **(1 specimen + 1 NC)**		
**DNA extraction**	13.44	25.76	/	/
**PCR**	2.04	3.91	/	/
**Library preparation& sequencing**	53.39	188.56	/	/
**Total**	**68.87**	**218.23**	**514.84**	**321.77**

Our method had several limitations. First, the sensitivity for low-abundance microbial species was lower in a background of upper respiratory commensals. Second, we observed that 27F primer failed to detect *C. pneumoniae* for Patient 12, 20 and 28. In silico sequence evaluation revealed mismatches at 3′ end of 27F primer when aligned with *C. pneumoniae* reference sequence (nucleotide 8–20, NCBI Accession Number: NR_026527.1). The problem was resolved by including 533F-1100R primers for 16S analysis and this highlighted the importance of optimal primer choices for the best species coverage. We also observed that several species were misidentified by EPI2ME, for instance, *Streptococcus mitis* was misidentified as *S. pneumoniae*, and *C. albicans* was misidentified as *Candida auris*. Confirmation of results with alternative pipelines is therefore advisable. Like many DNA-based molecular assays, we could not determine cell viability from sequencing data. Close communication between physicians and laboratorians is therefore warranted to correlate laboratory data with clinical presentation. In addition, as this was purely a laboratory microbiology study, we had no clinical outcome data on treatment results to validate the etiology of each case.

## Conclusion

Our data shed light on potential value of targeted Nanopore sequencing for supplementing the limitations of conventional diagnostic workflow. It may not have the best of its value for common non-viral causes of respiratory infection, as these can be readily resolved by microbial culture and multiplex PCR, yet it may contribute to improved etiological diagnosis of opportunistic infection caused by rare pathogens. Future prospective study with greater pathogen diversity and clinical outcome data is warranted to further assess assay performance and streamline the protocol. With continuously improving sequencing technology as well as user-friendly workflow and price, we envision that Nanopore-based methods will be closer to the doors of clinical laboratories in the near future.

## Data Availability

The datasets used and/or analyzed during the current study are available from the corresponding author on reasonable request.

## Abbreviations

BLAST: Basic local alignment search tool
CAP: Community-acquired pneumonia
DNA: Deoxyribonucleic acid
FDA: Food and drug administration
ITS: Internal transcribed spacer
LRTI: Lower respiratory tract infection
NCBI: National center for biotechnology information
NGS: Next-generation sequencing
PCR: Polymerase chain reaction
rRNA: Ribosomal ribonucleic acid
WIMP: ‘What’s in my Pot?’
